# Supplementary material for: The dual role of Spn-E in supporting heterotypic ping-pong piRNA amplification in silkworms
Source: EMBO Rep. 2024 Apr 17;25(5):11. doi: 10.1038/s44319-024-00137-2 (PMC11094040; doi:10.1038/s44319-024-00137-2)
Supplement: Supplementary file 1 — Table EV1 [file 44319_2024_137_MOESM1_ESM.pdf]

Table EV1. Oligonucleotide sequences used in this study.

Plasmid construction	5′- 3′
Spn-E-E251Q-F	TCTTGATCAAGTTCACGAAAGAGGACAAGAGATG
Spn-E-E251Q-R	GTGAACTTGATCAAGAATAACATGGGTGTATTC
FLAG-DDX43-F	ATGACGACGATAAGACTGATCGCGACGATGATGA
pIEx1-DDX43-R	CTGAGGTTAATCACTTACCATCTGCCTCTCCCTCTAC
FLAG-R	CTTATCGTCGTCATCCTTGTAATC
pIEx-3'out-F	GTGATTAACCTCAGGTTATAC
HA-DDX43-F	GTTCCAGATTACGCTACTGATCGCGACGATGATGA
HA-R	AGCGTAATCTGGAACATCGTAT
DDX43-D399A-F	GTTTTAGCTGAGGCAGATAGGATGTTAGACATG
DDX43-D399A-R	TGCCTCAGCTAAAACAATATATGAGAAATTTATG
pFastBac-5'out-R	GGTTTCGGACCGAGATCCG
pFastBac-3'out-F	AAGCTTGTCGAGAAGTACTAG
pFastBac-6HFLAG-F	TCTCGGTCCGAAACCATGCATCATCATCATCATCATGATTACAAGGATGACGACGAT
pFastBac-Spn-E-R	TTCTCGACAAGCTTTTATGTTTGGAACATCTGAAAATC
SBP-Spn-E-F	GCCAGCGGGAGCCCGATGAATTAAAAGCATTCTTCAATTC
pcDNA5-Spn-E-R	GCATTACTTAGCTATGTTTGGAACATCTGAAAATCT
pCold-3'out-F	TAGGTAATCTCTGCTTAAAAGC
pCold-5'out-R	CCTACCTTCGATATGATGATG
pCold-DDX43-F	ATATCGAAGGTAGGACTGATCGCGACGATGATG
pCold-DDX43-R	GCAGAGATTACCTACCATCTGCCTCTCCCTCT
pCold-DDX6-F	ATATCGAAGGTAGGATGACCGAAAATAGAATTAGTTC
pCold-DDX6-R	AGCAGAGATTACCTACTTGTCGCCCAGATCTTC
piR484-A_s	GATCCTACCAATCGGGCGTTGCTGTTGCAATTGAAGGAATGAAGACGAAGAAAGTATTGATGAACAGCACAATA
piR484-A_as	AGCTTATTGTGCTGTTCATCAATACTTTCTTCGTCTTCATTCCTTCAATTGCAACAGCAACGCCCGATTGGTAG
pIB-BmAgo3_PiggyBac_F	TATCAATCGGGCGTTGCTGTTGCAATTCGGTCTCGATTCTACGC
pIB-BmAgo3_PiggyBac_R	AATTGCAACAGCAACGCCCGATTGATAAGGAGAGGGTTAGGGATAGGCTTAC
**Target RNA preparation**	
BmAgo3 target RNA 5' fragment	AGAGUCCUUCGAUAGGGACAAGACAAUUGCACUGAUCAUGAACUCCUCUCUUCCCGCAGACAGCAAAUUCUCA
BmAgo3 target RNA 3' fragment	UGCUUUUCCUUUUAUACAACCGUUCUACACUCAACGCGAUGUAAAUC
BmAgo3 target bridge oligo	TATAAAAGGAAAAGCATGAGAATTTGCTGTC
**dsRNA preparation**	
dsRluc (*Renilla luciferase*) -F	TAATACGACTCACTATAGGGCCTTTCACTACTCCTACGAGC
dsRluc (*Renilla luciferase*) -R	TAATACGACTCACTATAGGGTGGAGCGTCCTCCTGGCTG
dsSiwi-F	GCGTAATACGACTCACTATAGGATCACCCCAGAAAGACAACG
dsSiwi-R	GCGTAATACGACTCACTATAGGCTGTGCACGTATGGGATTTG
dsSpnE-CDS-F	TAATACGACTCACTATAGGGACCGCAAGATCATTCTTTCCA
dsSpnE-CDS-R	TAATACGACTCACTATAGGGTCCGTAGACATAGCCGAGCA
dsSpnE-UTR-F	TAATACGACTCACTATAGGGACCGTTTATTTTTAACATAACTG
dsSpnE-UTR-R	TAATACGACTCACTATAGGGGCCATATTTTCAATTTCACTTC
dsDDX43#1-F	TAATACGACTCACTATAGGGACCGATAAAGTAGGTAGGG
dsDDX43#1-R	TAATACGACTCACTATAGGGCAGCTTATCCCATCTGTTGT
dsDDX43#2-F	TAATACGACTCACTATAGGGACATGGCCTACTGGAGTAC
dsDDX43#2-R	TAATACGACTCACTATAGGGATGCAACATCAGTGGCAATC
dsDDX6-F	TAATACGACTCACTATAGGGCGTGTGTTTCACGACTTTCG
dsDDX6-R	TAATACGACTCACTATAGGGTTGGGAATTGGCTTGATTTC
**Quantitative real-time PCR**	
rp49 -F	AGGTATTGACAACAGAGTCC
rp49 -R	GGAGCATATGACGGGTCTTC
DDX43 -F	GTAGTCGTACAGACACCGGC
DDX43 -R	TATTACGGCCGCGATTTCCA
**Probes for northern blot analysis**	
piR484-A	TCATTCCTTCAATTGCAACA
Artificial_Siwi_piRNA_PiggyBac	TACGCGTAGAATCGAGACCGAATTGCAACA
