# Supplementary figures and images for: The dual role of Spn-E in supporting heterotypic ping-pong piRNA amplification in silkworms
Source: EMBO Rep. 2024 Apr 17;25(5):11. doi: 10.1038/s44319-024-00137-2 (PMC11094040; doi:10.1038/s44319-024-00137-2)

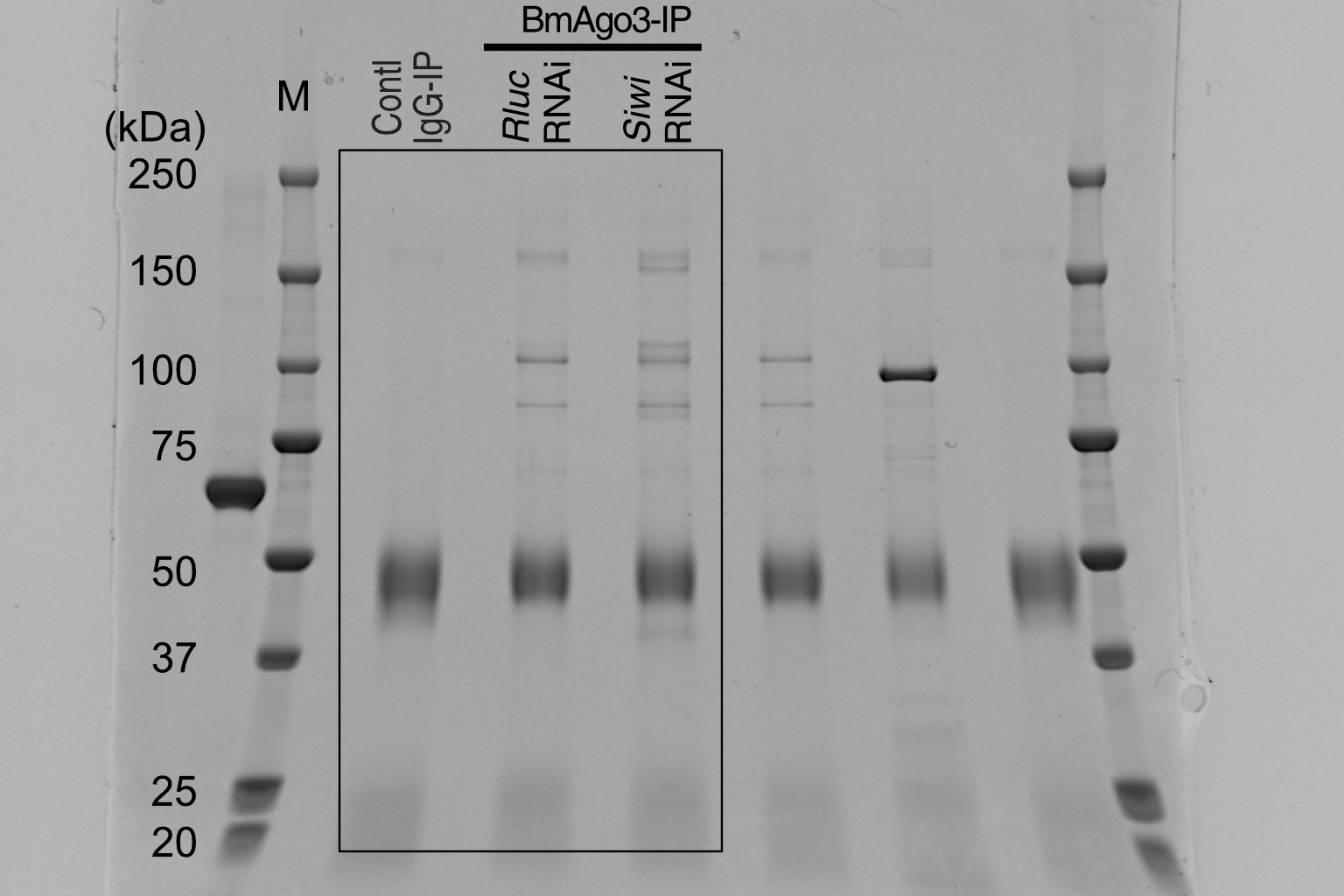

Supplement: Supplementary file 2 — Source data Fig. 1 [file 44319_2024_137_MOESM2_ESM.zip › Figure 1/1A/CBB stain1.tiff]

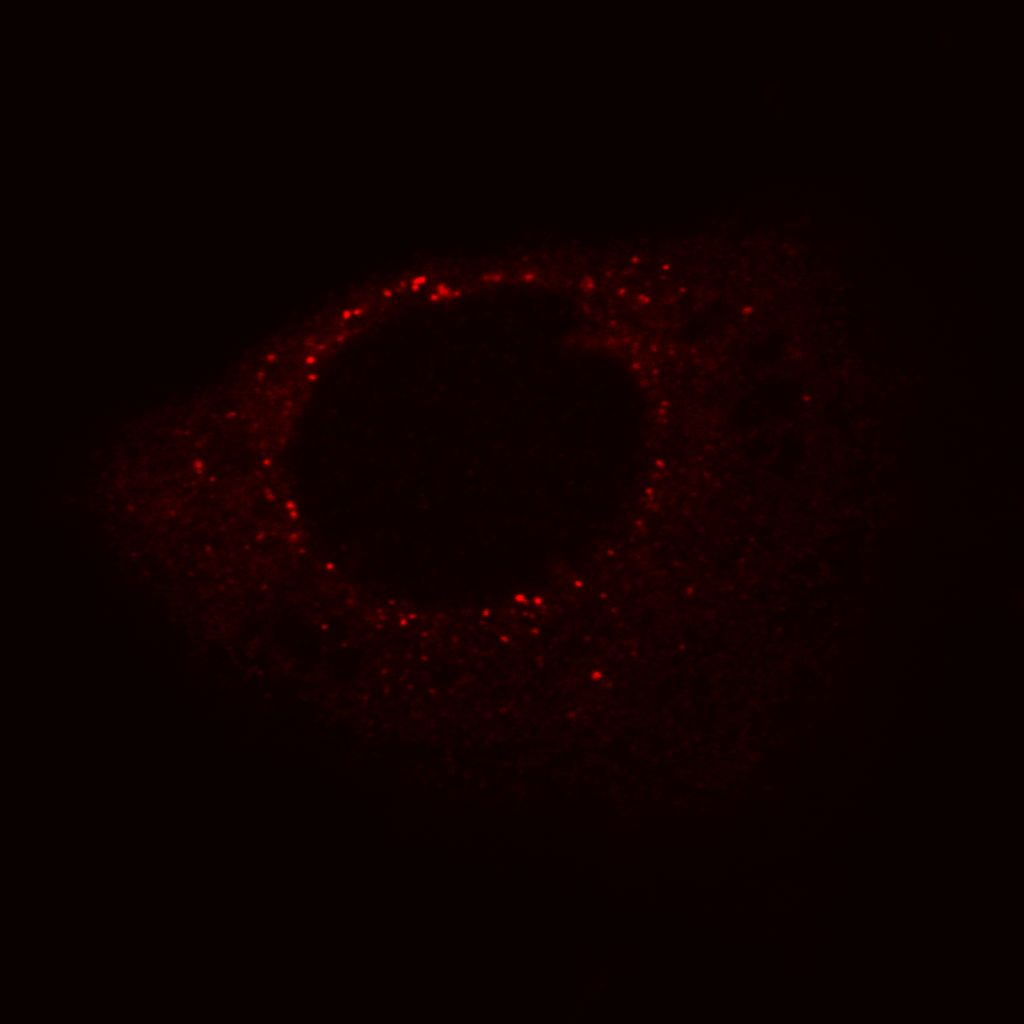

Supplement: Supplementary file 2 — Source data Fig. 1 [file 44319_2024_137_MOESM2_ESM.zip › Figure 1/1B/Rluc RNAi/BmAgo3.tif]

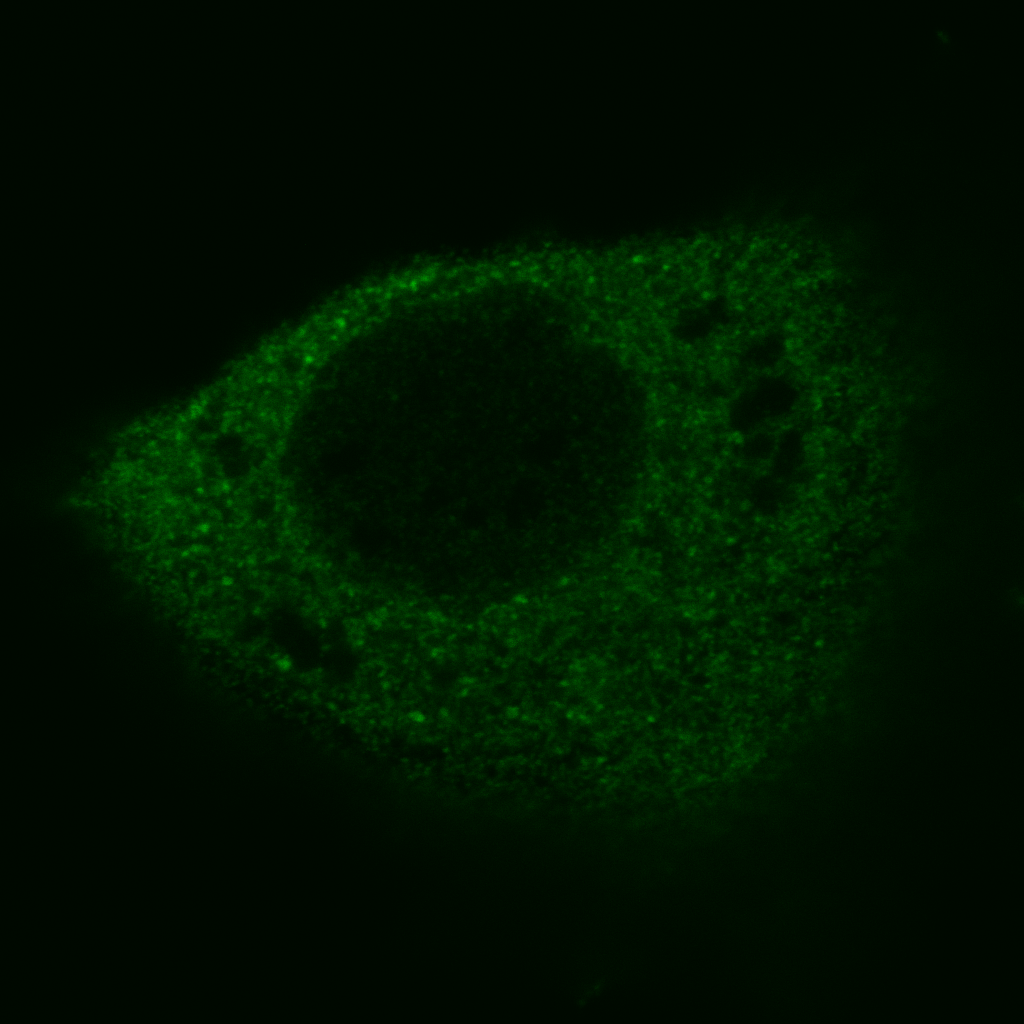

Supplement: Supplementary file 2 — Source data Fig. 1 [file 44319_2024_137_MOESM2_ESM.zip › Figure 1/1B/Rluc RNAi/FLAG-DDX32.tif]

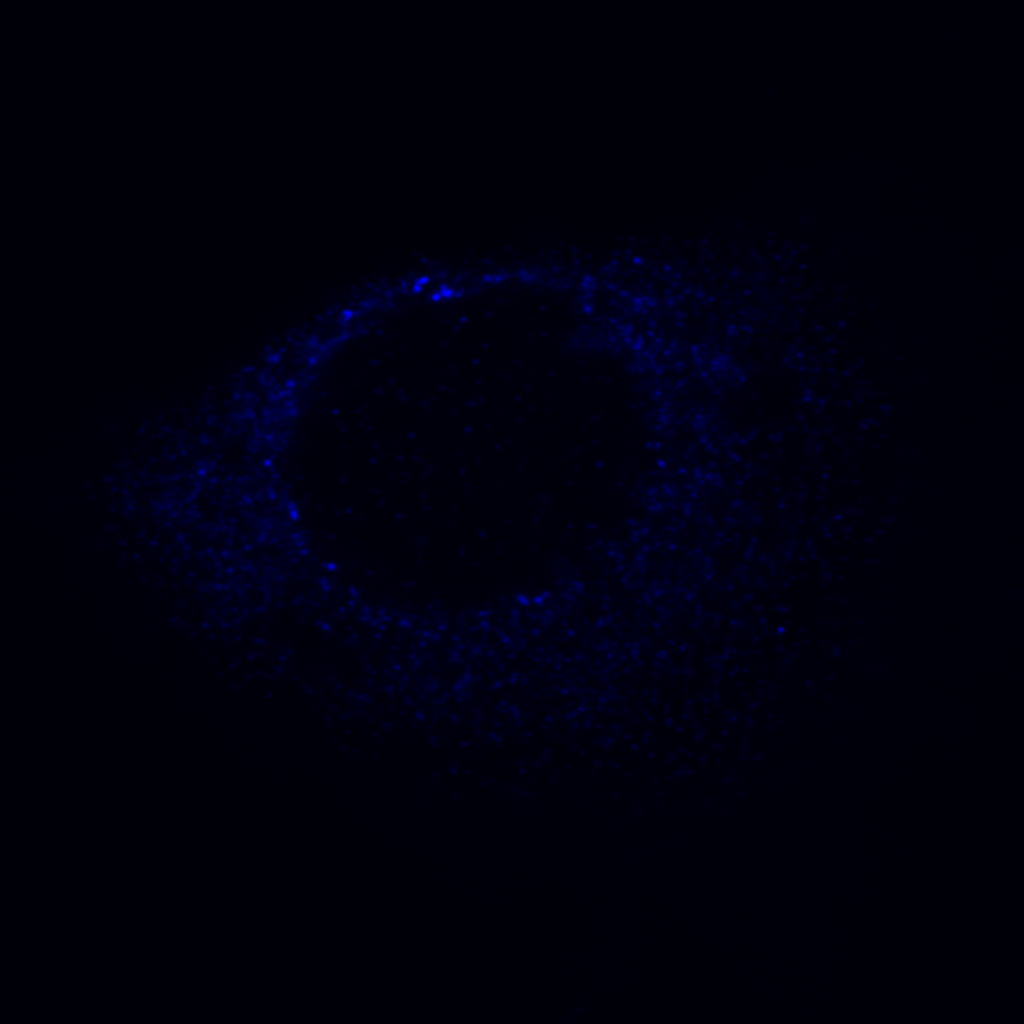

Supplement: Supplementary file 2 — Source data Fig. 1 [file 44319_2024_137_MOESM2_ESM.zip › Figure 1/1B/Rluc RNAi/Spn-E.tif]

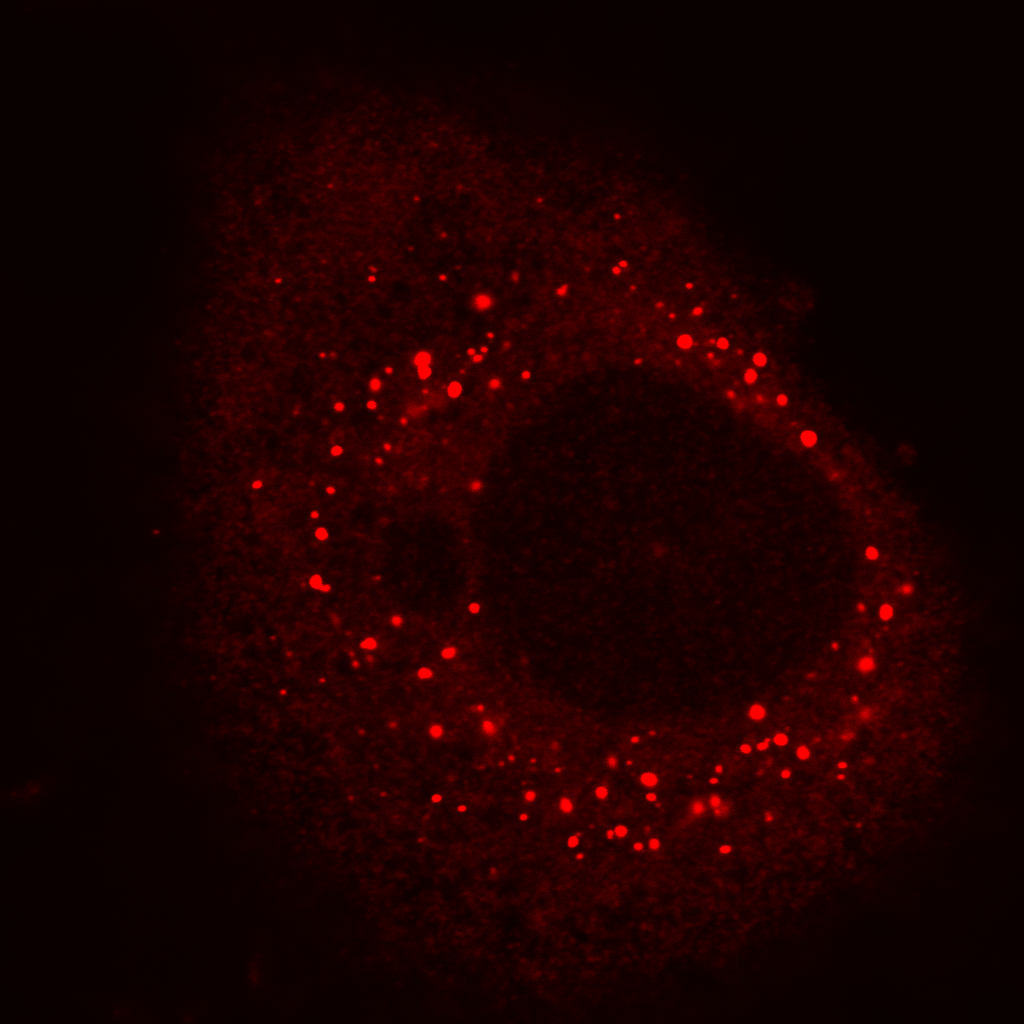

Supplement: Supplementary file 2 — Source data Fig. 1 [file 44319_2024_137_MOESM2_ESM.zip › Figure 1/1B/Siwi RNAi/BmAgo3.tif]

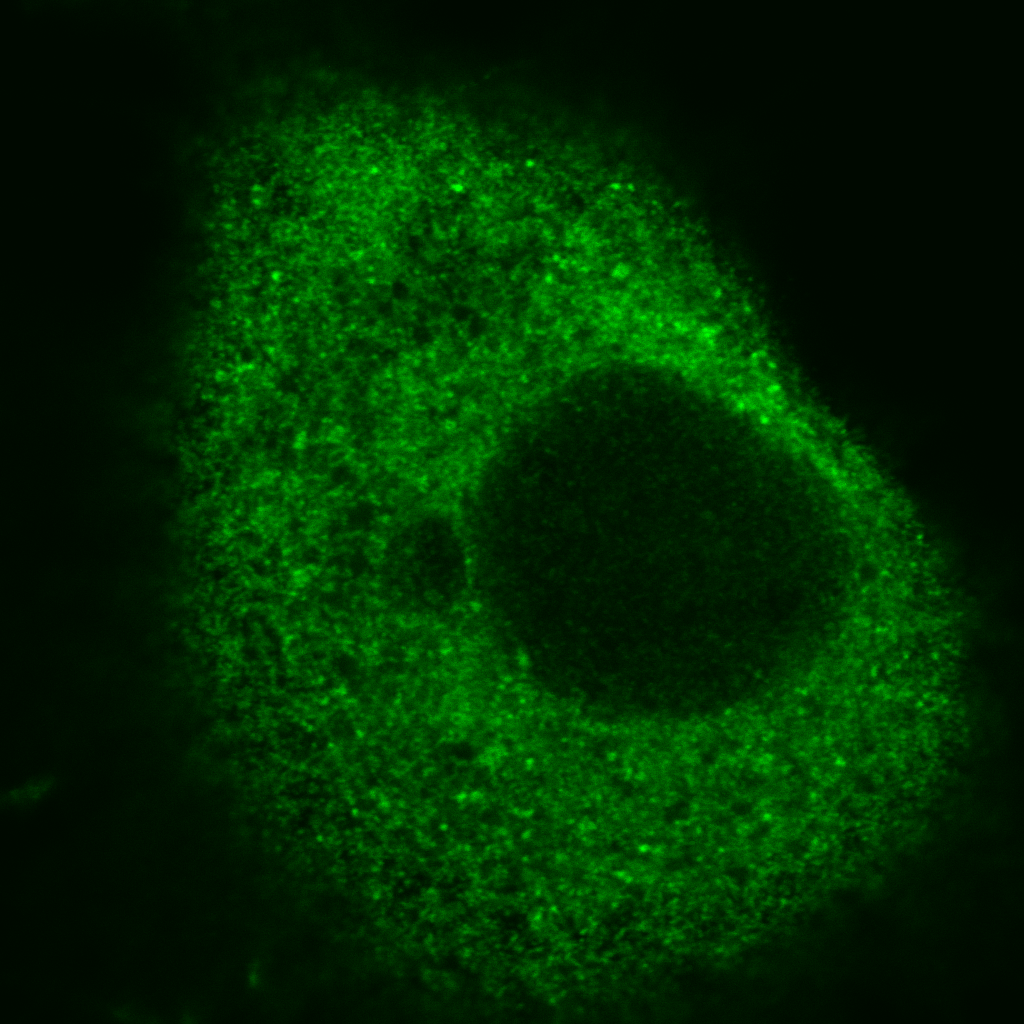

Supplement: Supplementary file 2 — Source data Fig. 1 [file 44319_2024_137_MOESM2_ESM.zip › Figure 1/1B/Siwi RNAi/FLAG-DDX43.tif]

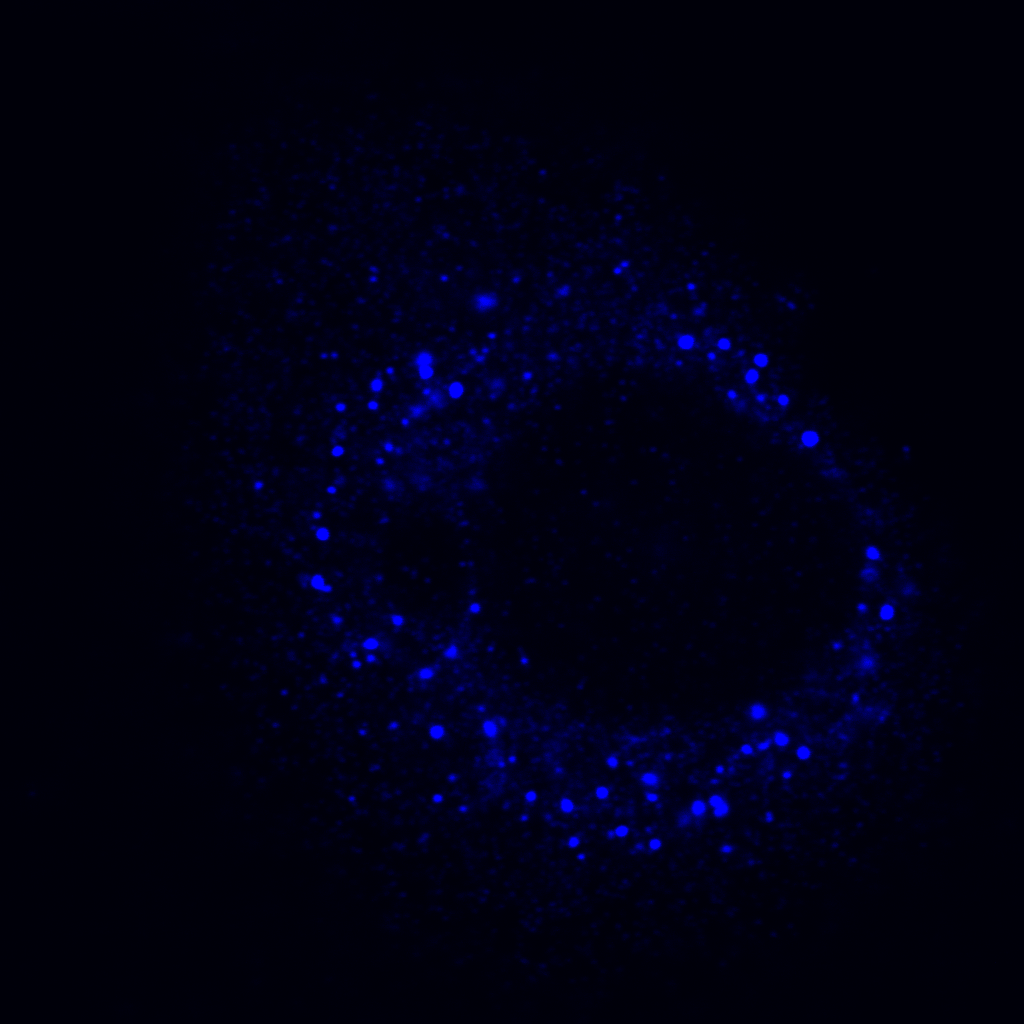

Supplement: Supplementary file 2 — Source data Fig. 1 [file 44319_2024_137_MOESM2_ESM.zip › Figure 1/1B/Siwi RNAi/Spn-E.tif]

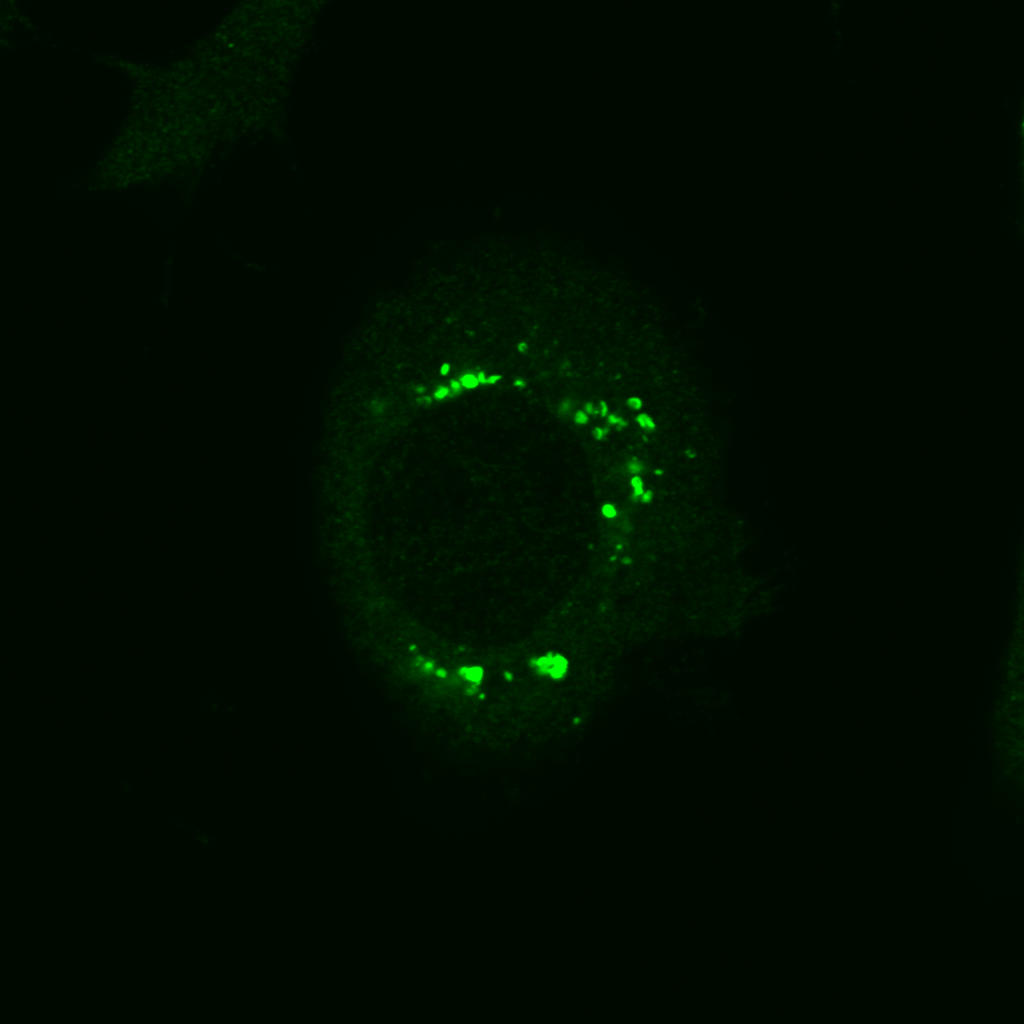

Supplement: Supplementary file 2 — Source data Fig. 1 [file 44319_2024_137_MOESM2_ESM.zip › Figure 1/1C/Spn-E-EQ/BmAgo3.tif]

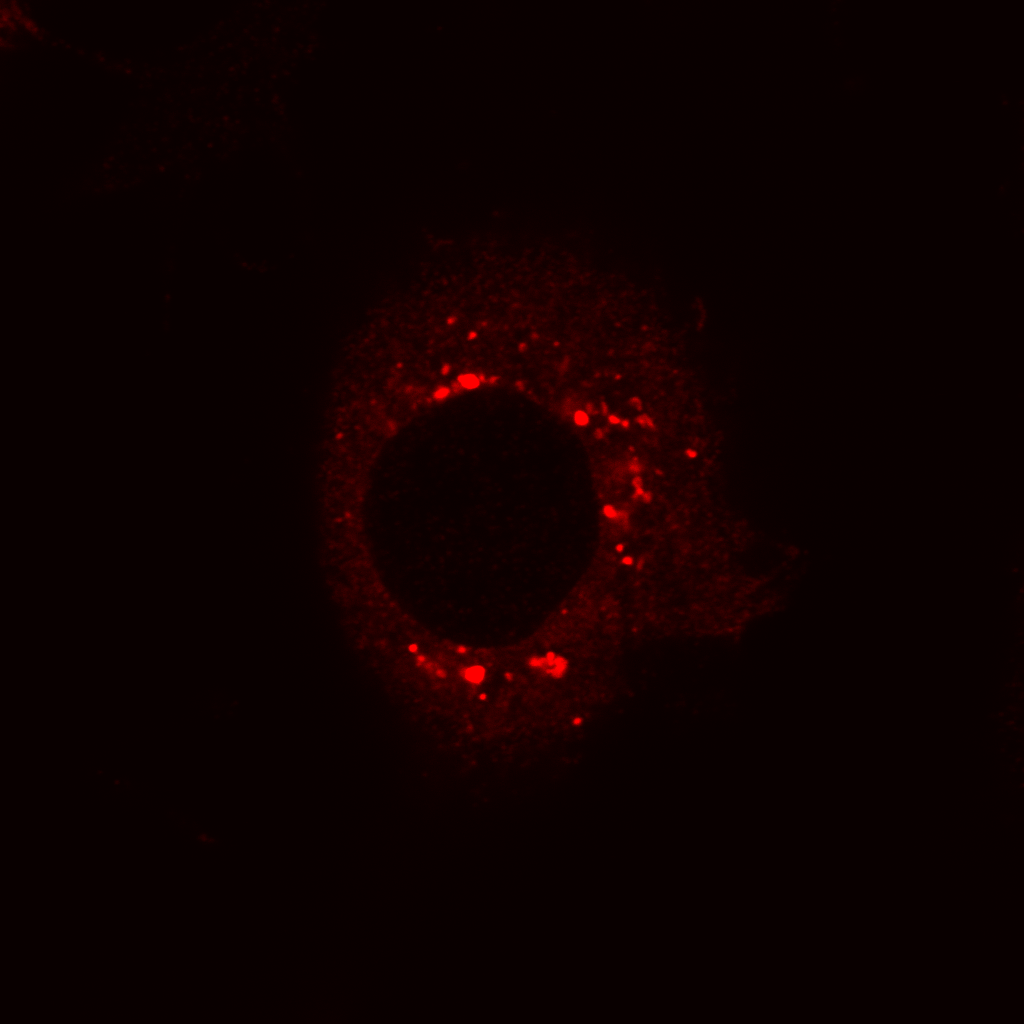

Supplement: Supplementary file 2 — Source data Fig. 1 [file 44319_2024_137_MOESM2_ESM.zip › Figure 1/1C/Spn-E-EQ/FLAG-Spn-E-EQ.tif]

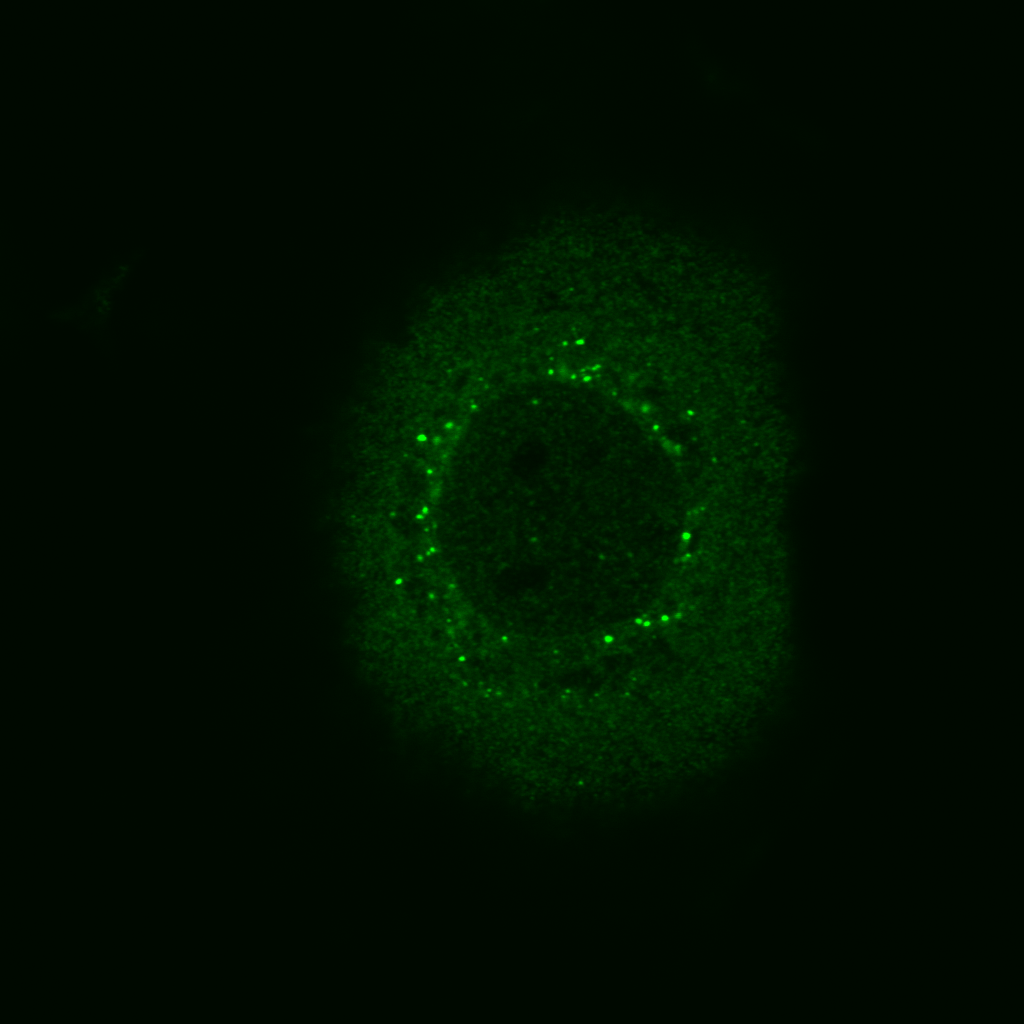

Supplement: Supplementary file 2 — Source data Fig. 1 [file 44319_2024_137_MOESM2_ESM.zip › Figure 1/1C/Spn-E-WT/BmAgo3.tif]

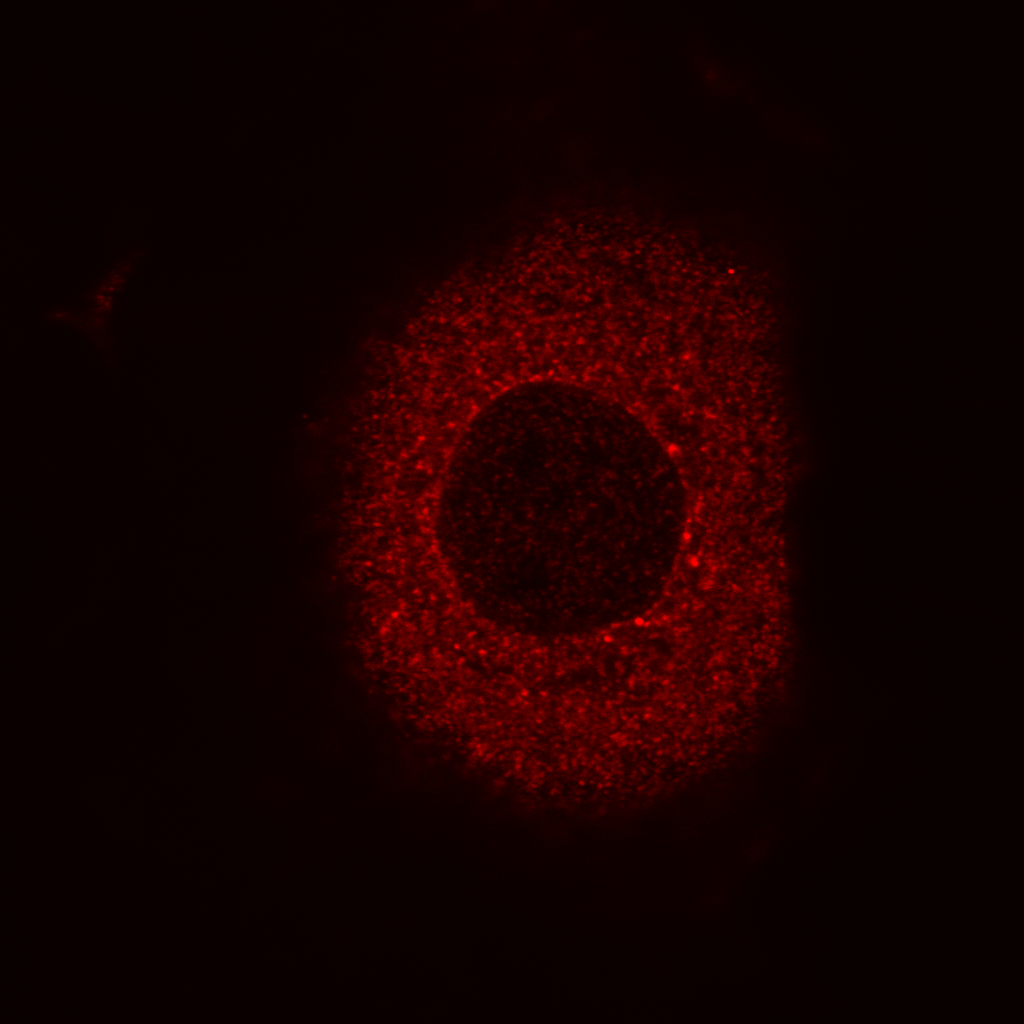

Supplement: Supplementary file 2 — Source data Fig. 1 [file 44319_2024_137_MOESM2_ESM.zip › Figure 1/1C/Spn-E-WT/FLAG-Spn-E-WT.tif]

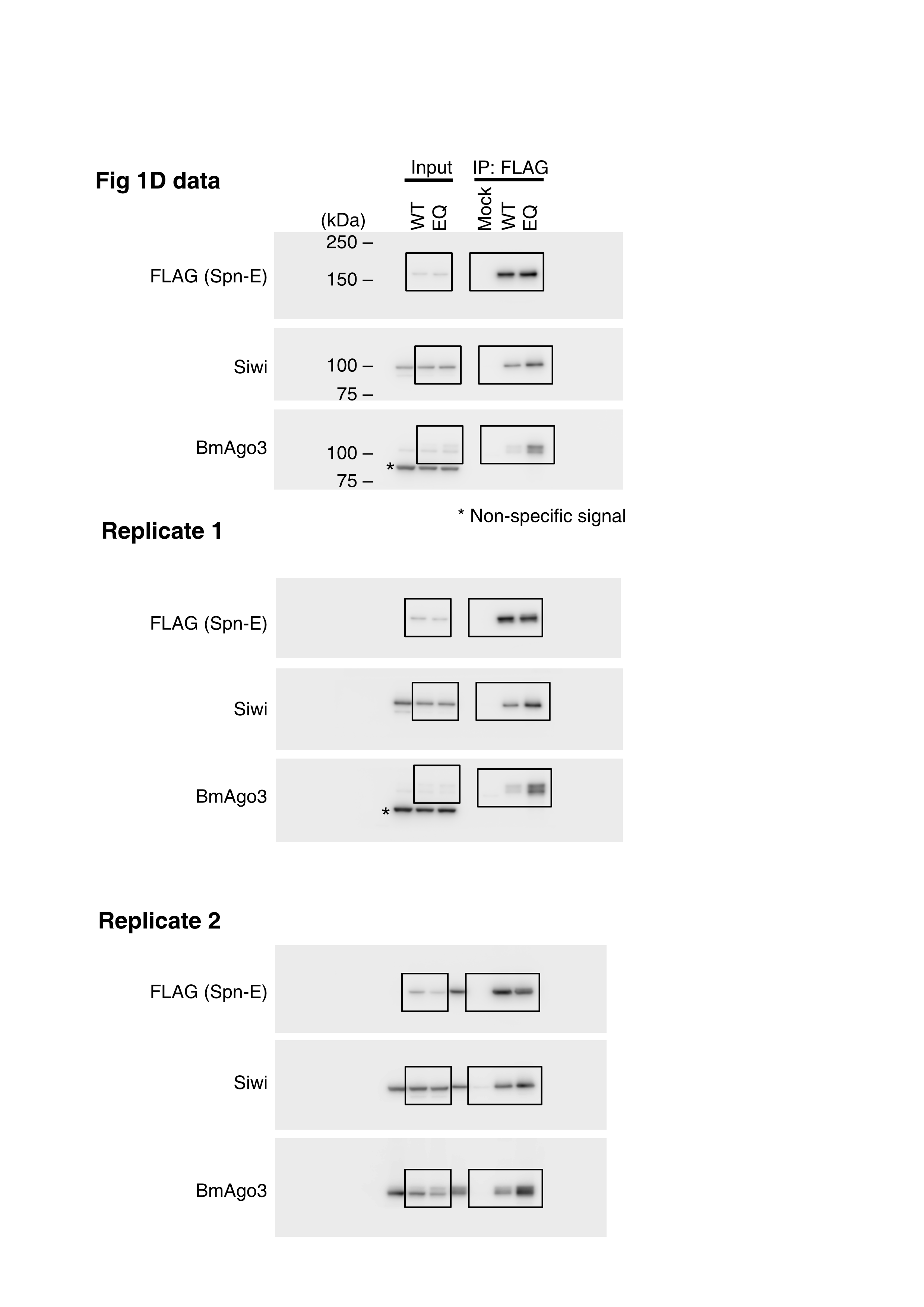

Supplement: Supplementary file 2 — Source data Fig. 1 [file 44319_2024_137_MOESM2_ESM.zip › Figure 1/1D/IP-WB.tiff]

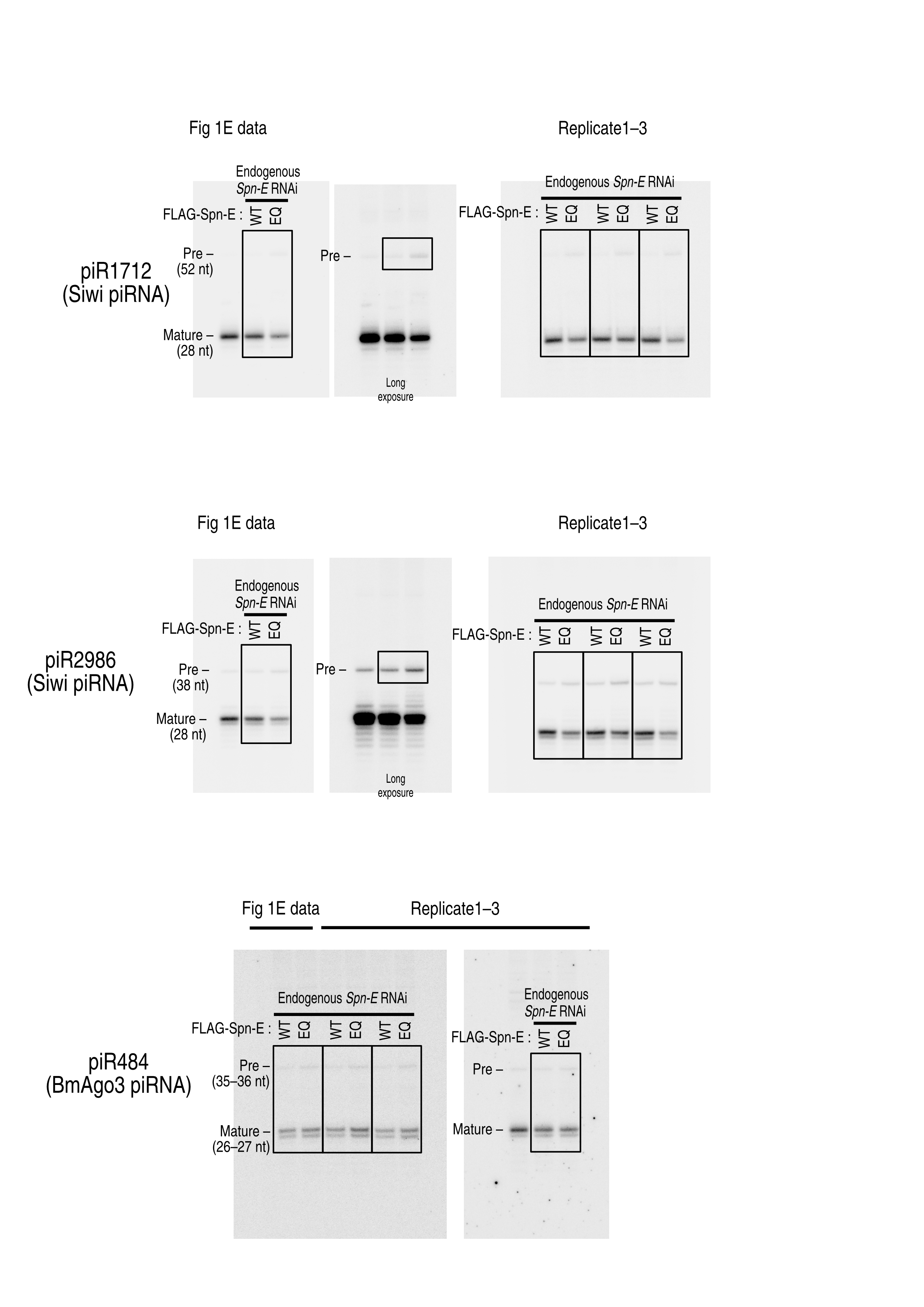

Supplement: Supplementary file 2 — Source data Fig. 1 [file 44319_2024_137_MOESM2_ESM.zip › Figure 1/1E/NB_4exp.tiff]

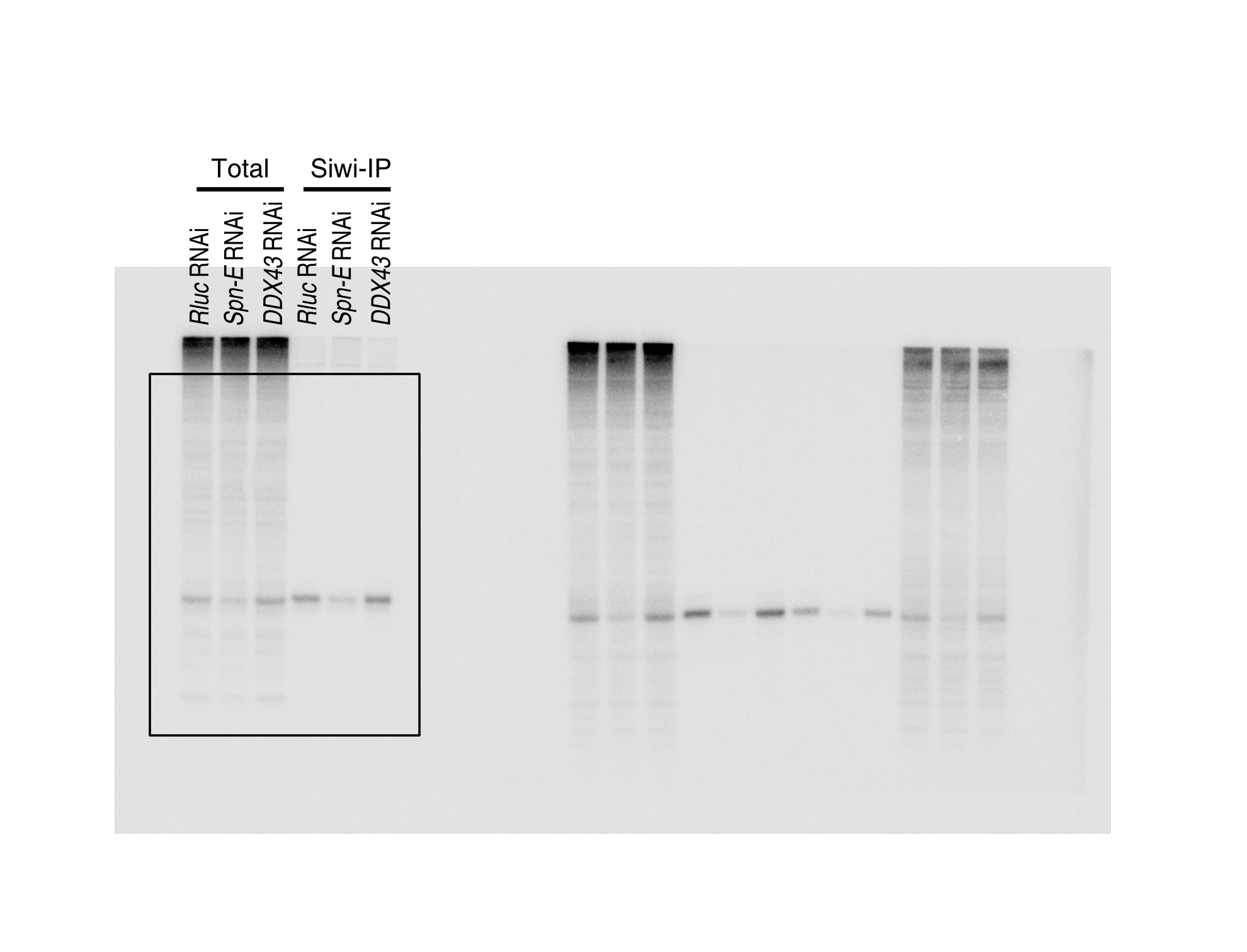

Supplement: Supplementary file 4 — Source data Fig. 3 [file 44319_2024_137_MOESM4_ESM.zip › Figure 3/3B/NB_piR484-A/230721_NB_art reporter_piR484A.tiff]

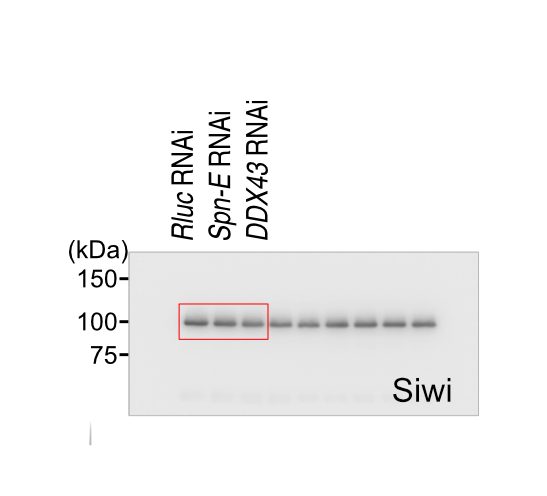

Supplement: Supplementary file 4 — Source data Fig. 3 [file 44319_2024_137_MOESM4_ESM.zip › Figure 3/3B/WB_Siwi/230713_PB_SwIPw2_pi484A.tiff]

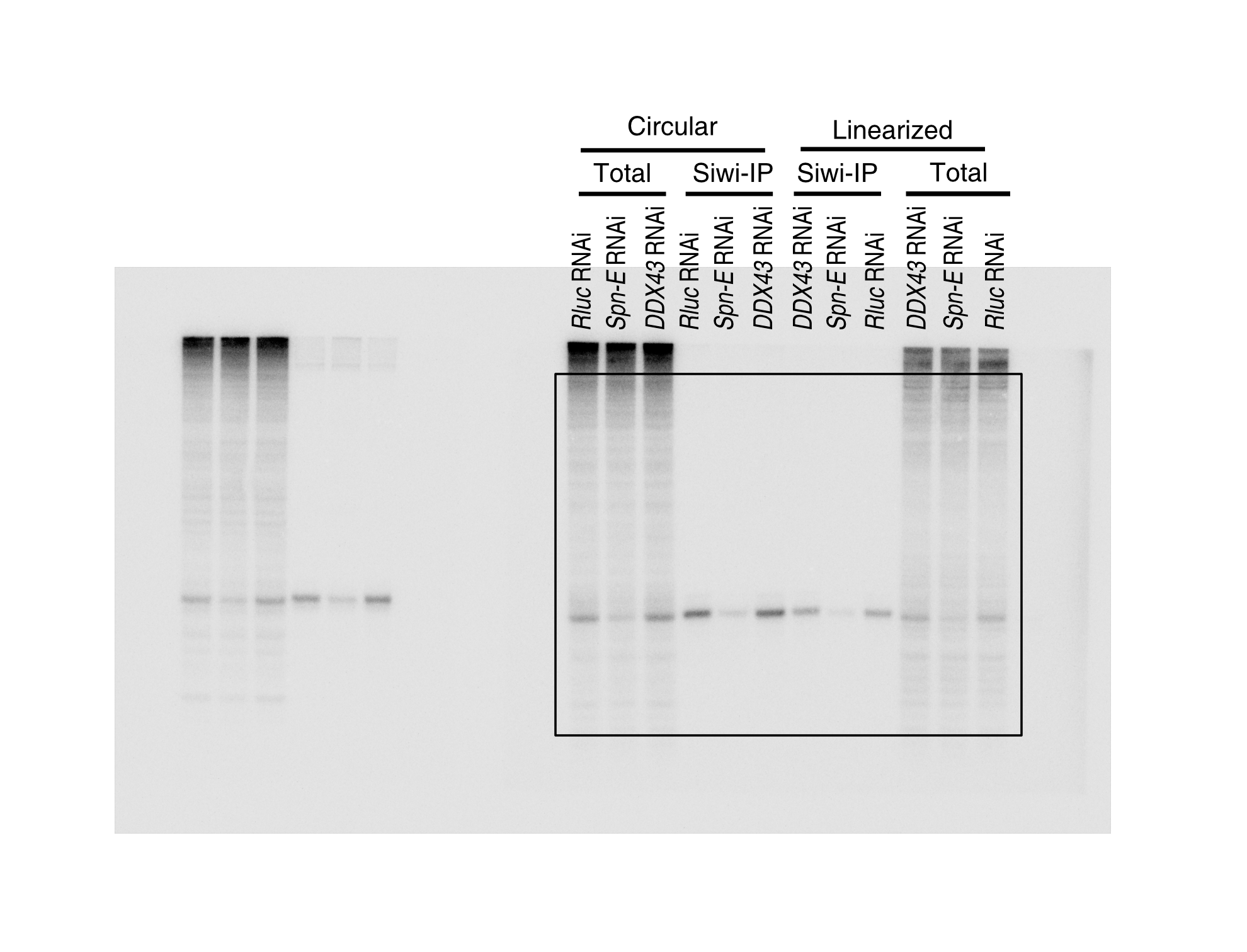

Supplement: Supplementary file 4 — Source data Fig. 3 [file 44319_2024_137_MOESM4_ESM.zip › Figure 3/3D/NB_art-Siwi piRNA/230721_NB_art reporter_PB.tiff]

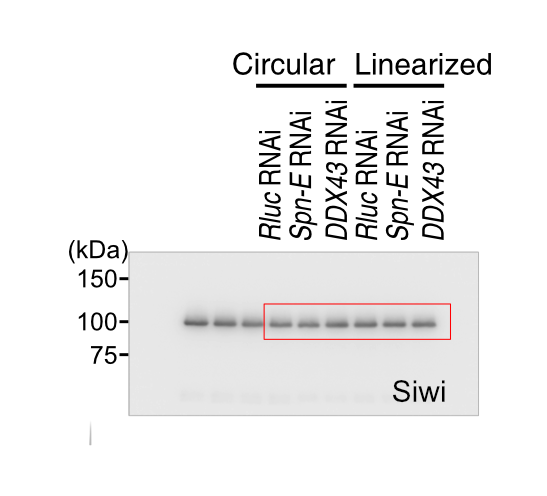

Supplement: Supplementary file 4 — Source data Fig. 3 [file 44319_2024_137_MOESM4_ESM.zip › Figure 3/3D/WB_Siwi/230713_PB_SwIPw2.tiff]

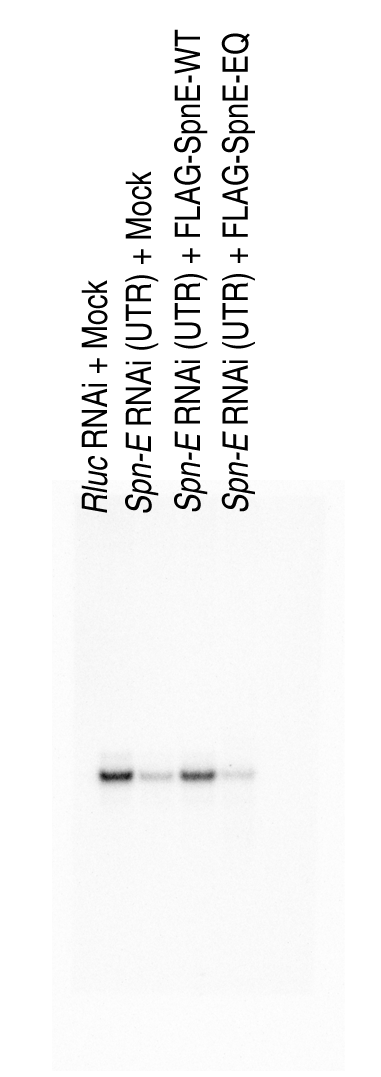

Supplement: Supplementary file 4 — Source data Fig. 3 [file 44319_2024_137_MOESM4_ESM.zip › Figure 3/3E/NB_art-Siwi piRNA/231016_PB reporter_3on.tif]

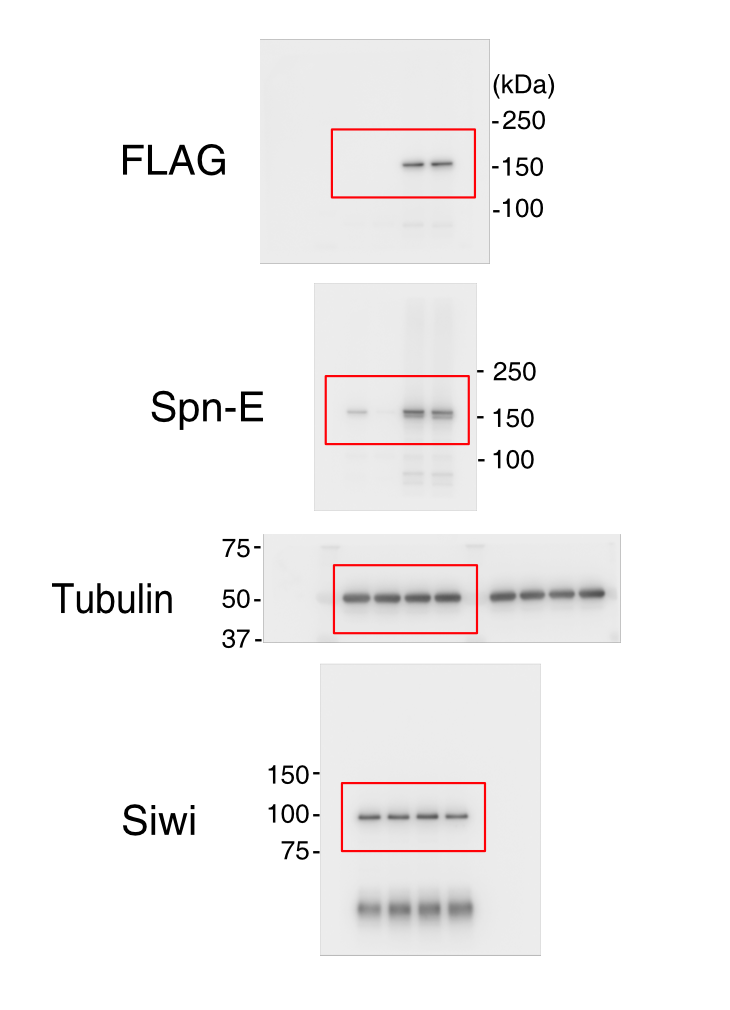

Supplement: Supplementary file 4 — Source data Fig. 3 [file 44319_2024_137_MOESM4_ESM.zip › Figure 3/3E/WB/231014_3_WB.tiff]
