# Supplementary material for: The dual role of Spn-E in supporting heterotypic ping-pong piRNA amplification in silkworms
Source: EMBO Rep. 2024 Apr 17;25(5):11. doi: 10.1038/s44319-024-00137-2 (PMC11094040; doi:10.1038/s44319-024-00137-2)
Supplement: Supplementary file 6 — Expanded View Figures [file 44319_2024_137_MOESM6_ESM.pdf]

Expanded View Figures

Figure EV1. Characterization of Spn-E-EQ aggregates and BmAgo3-cleavage fragment release assay.

(**A**) Quantification of Siwi and BmAgo3 signals co-immunoprecipitated with FLAG-Spn-E (wild type or EQ) in Fig. 1D. Relative co-immunoprecipitated levels normalized to the Spn-E IP level are shown. Data are mean ± s.d. of three independent experiments (biological replicates). Statistical analysis was performed using a two-sided Student's paired *t*-test, with *p*-values adjusted using the Holm method. NS not significant. (**B**) Western blot analysis of whole cell lysates of BmN4 cells treated with dsRNA for *Rluc* (control) or *DDX6*. The anti-DDX6 antibody successfully detected endogenous DDX6. (**C**) Subcellular localization of FLAG-Spn-E-EQ, DDX6, a P-body marker protein, and BmAgo3 in BmN4 cells. Scale bar, 5 µm. (**D**) Subcellular localization of FLAG-Spn-E-EQ, BmAgo3 and Siwi in BmN4 cells. Scale bar, 5 µm. (**E**) Tandem IP experiment on BmN4 cells co-transfected with the FLAG-Spn-E-EQ expression plasmid and dsRNA targeting the *Spn-E* 3′ UTR. FLAG-Spn-E-EQ was first immunoprecipitated with the FLAG tag, and the resulting immunopurified complex was then subjected to a second IP with normal rabbit IgG (Contl-IgG) or an anti-BmAgo3 antibody. Siwi in the second immunoprecipitate was detected by western blotting with or without RNase A treatment. (**F**) Quantification of mature piRNA signals in Fig. 1E. Relative expression levels normalized to those under Spn-E-WT expression are shown. Data are mean ± s.d. of four independent experiments (biological replicates). Statistical analysis was performed using a two-sided Student's paired *t*-test, with *p*-values adjusted using the Holm method ($*p = 0.023$). (**G**) Quantification of pre-piRNA signals in Fig. 1E. Relative expression levels normalized to those under Spn-E-WT expression are shown. Data are mean ± s.d. of four independent experiments (biological replicates). Statistical analysis was performed using a two-sided Student's paired *t*-test, with *p*-values adjusted using the Holm method ($*p = 0.025$, $**p = 0.003$). (**H**) CBB staining of purified recombinant Spn-E and DDX43 proteins (rSpn-E and rDDX43, indicated by arrowheads) used for the in vitro cleavage fragment release assay. (**I**) Top: Schematic representation of the the in vitro cleavage fragment release assay. To detect cleaved 5′ and 3′ fragments separately, the target RNA was radiolabeled at different positions (*). The 5′ or internally radiolabeled target RNAs were subjected to a cleavage assay using BmAgo3 immunoprecipitates. After the reaction, the bead fraction was incubated with rSpn-E or rDDX43 in the presence of ATP. Bottom: The cleaved fragments in the supernatant and bead fractions were detected by autoradiography. (**J**) Thin-layer chromatography for the detection of ATPase activity of the recombinant Spn-E protein. (**K**) Subcellular localization of FLAG-DDX43 (wild type or DA, an ATPase-deficient mutant) and BmAgo3 in BmN4 cells. Scale bar, 5 µm. (**L**) Subcellular localization of FLAG-Spn-E-EQ, HA-DDX43, and BmAgo3 in BmN4 cells. Scale bar, 5 µm.

© The Author(s)

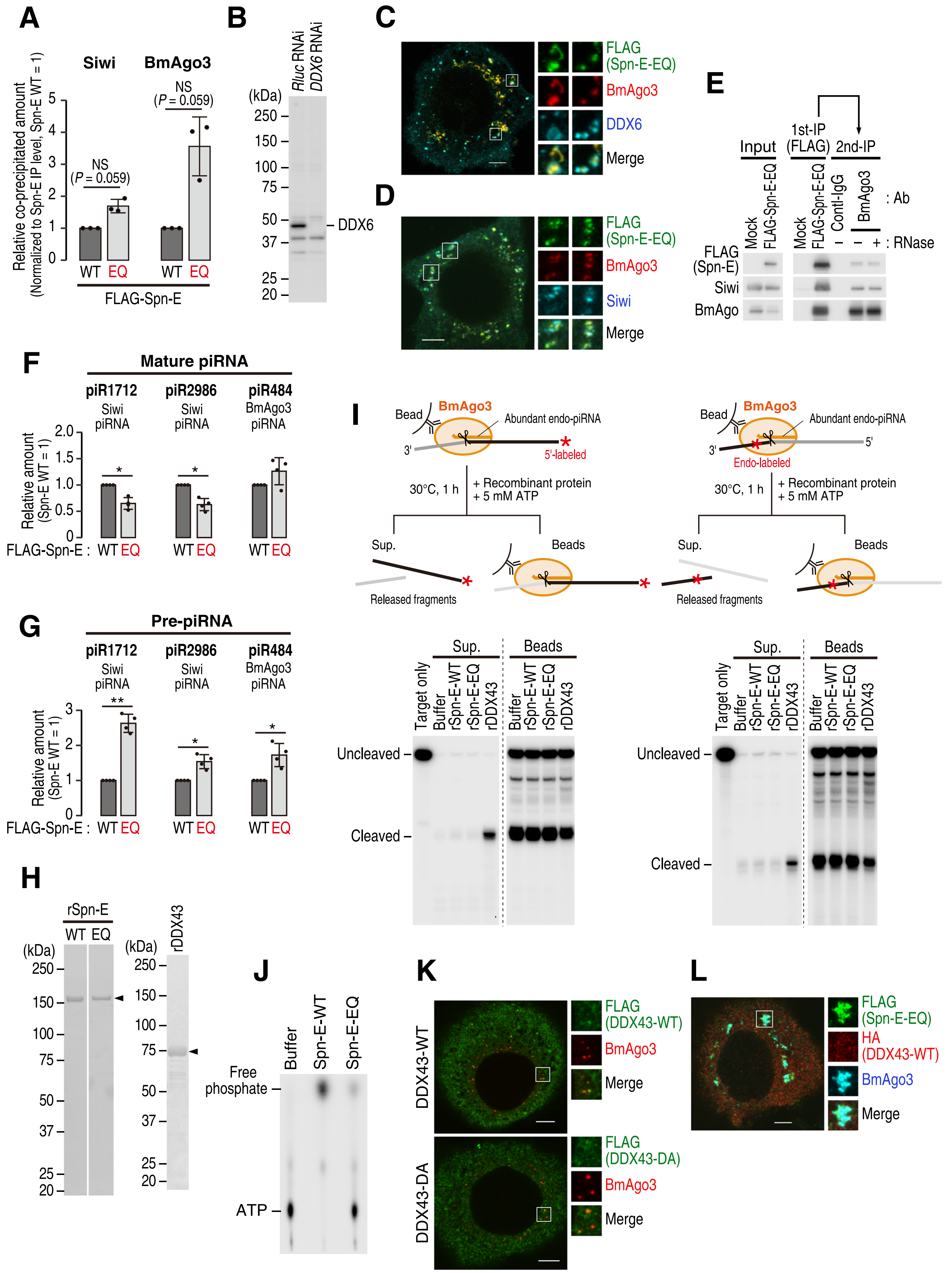

A
Siwi
BmAgo3
Relative co-precipitated amount (Normalized to Spn-E IP level, Spn-E WT = 1)
NS (P = 0.059)
NS (P = 0.059)
WT EQ WT EQ
FLAG-Spn-E
B
Rluc RNAi
DDX6 RNAi
(kDa)
DDX6
C
FLAG (Spn-E-EQ)
BmAgo3
DDX6
Merge
D
FLAG (Spn-E-EQ)
BmAgo3
Siwi
Merge
E
Input
1st-IP (FLAG)
2nd-IP
Mock
FLAG-Spn-E-EQ
Contl-IgG
BmAgo3
: Ab
: RNase
FLAG (Spn-E)
Siwi
BmAgo
F
Mature piRNA
piR1712 Siwi piRNA
piR2986 Siwi piRNA
piR484 BmAgo3 piRNA
Relative amount (Spn-E WT = 1)
FLAG-Spn-E :
G
Pre-piRNA
H
rSpn-E
WT EQ
rDDX43
(kDa)
I
Bead
BmAgo3
Abundant endo-piRNA
5'-labeled
Endo-labeled
30°C, 1 h
+ Recombinant protein
+ 5 mM ATP
Sup.
Beads
Released fragments
Target only
Buffer
rSpn-E-WT
rSpn-E-EQ
rDDX43
Uncleaved
Cleaved
J
Buffer
Spn-E-WT
Spn-E-EQ
Free phosphate
ATP
K
DDX43-WT
DDX43-DA
FLAG (DDX43-WT)
FLAG (DDX43-DA)
BmAgo3
Merge
L
FLAG (Spn-E-EQ)
HA (DDX43-WT)
BmAgo3
Merge

© The Author(s)

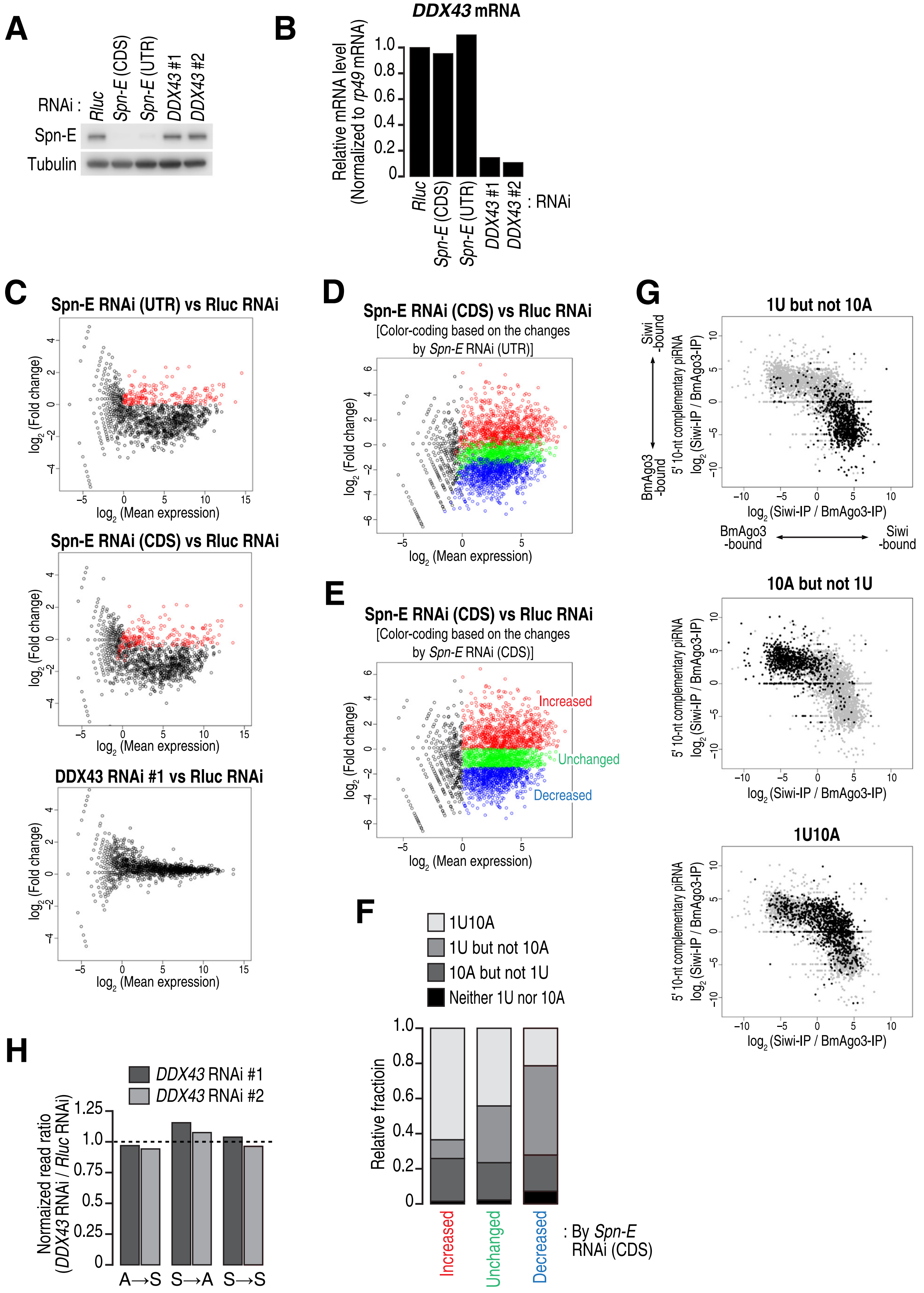
A
RNAi :
Rluc
Spn-E (CDS)
Spn-E (UTR)
DDX43 #1
DDX43 #2
Spn-E
Tubulin
B
DDX43 mRNA
Relative mRNA level (Normalized to rp49 mRNA)
: RNAi
C
Spn-E RNAi (UTR) vs Rluc RNAi
Spn-E RNAi (CDS) vs Rluc RNAi
DDX43 RNAi #1 vs Rluc RNAi
log2 (Fold change)
log2 (Mean expression)
D
Spn-E RNAi (CDS) vs Rluc RNAi
[Color-coding based on the changes by Spn-E RNAi (UTR)]
E
Spn-E RNAi (CDS) vs Rluc RNAi
[Color-coding based on the changes by Spn-E RNAi (CDS)]
Increased
Unchanged
Decreased
F
1U10A
1U but not 10A
10A but not 1U
Neither 1U nor 10A
Relative fractioin
: By Spn-E RNAi (CDS)
G
1U but not 10A
10A but not 1U
1U10A
Siwi -bound
BmAgo3 -bound
5' 10-nt complementary piRNA log2 (Siwi-IP / BmAgo3-IP)
log2 (Siwi-IP / BmAgo3-IP)
H
DDX43 RNAi #1
DDX43 RNAi #2
Normaized read ratio (DDX43 RNAi / Rluc RNAi)
A→S
S→A
S→S

 © The Author(s)

Figure EV2. Changes in the expression of TE-mapped piRNAs upon KD of *Spn-E* or *DDX43*.

(**A**) Western blot analysis of whole cell lysates from BmN4 cells treated with dsRNA for *Rluc* (control), *Spn-E*, or *DDX43*. Two different dsRNAs (*Spn-E*: CDS and 3′ UTR; *DDX43*: two different regions of the CDS, #1 and #2) were used for RNAi. *Rluc*; *Renilla luciferase*. (**B**) Quantitative real-time PCR analysis of the expression of *DDX43* in BmN4 cells treated with dsRNA for *Rluc* (control), *Spn-E*, or *DDX43*. Relative mRNA expression levels normalized to those of *rp49* are shown. *Rluc*; *Renilla luciferase*. (**C**) MA plots showing piRNA expression changes for each TE between the control KD (*Rluc* RNAi) and *Spn-E* or *DDX43* KD. Two different dsRNAs (CDS and 3′ UTR) were used for *Spn-E* RNAi. Each dot represents one TE. TEs with increased piRNA production in the *Spn-E* RNAi (UTR) are colored red. (**D**) MA plot showing piRNA expression changes between the control KD (*Rluc* RNAi) and *Spn-E* KD using dsRNA targeting the *Spn-E* CDS. Each dot represents one piRNA. Based on the three groups defined in Fig. 2A, piRNAs were color-coded as follows: "increased" (red, $n = 825$), "unchanged" (green, $n = 824$), and "decreased" (blue, $n = 825$). (**E**) MA plot showing piRNA expression changes between control KD (*Rluc* RNAi) and *Spn-E* KD using dsRNA targeting the *Spn-E* CDS. Each dot represents one piRNA. Based on the changes in expression, piRNAs were divided into three groups: "increased" (red, $n = 805$), "unchanged" (green, $n = 805$), and "decreased" (blue, $n = 805$). (**F**) Relative fractions of 1U10A, 1U but not 10A, 10A but not 1U, and neither 1U nor 10A piRNAs of each group in (**E**). (**G**) Scatter plots showing the PIWI binding bias of 1U but not 10A, 10A but not 1U, or 1U10A piRNAs (x-axis) and that of their putative partner piRNAs in the ping-pong cycle (*y* axis). (**H**) Changes in the expression of A → S, S → A, and S → S piRNAs in *DDX43* KD relative to control KD (*Rluc* RNAi). Two different dsRNAs targeting the *DDX43* CDS were used for RNAi. *Rluc*; *Renilla luciferase*.

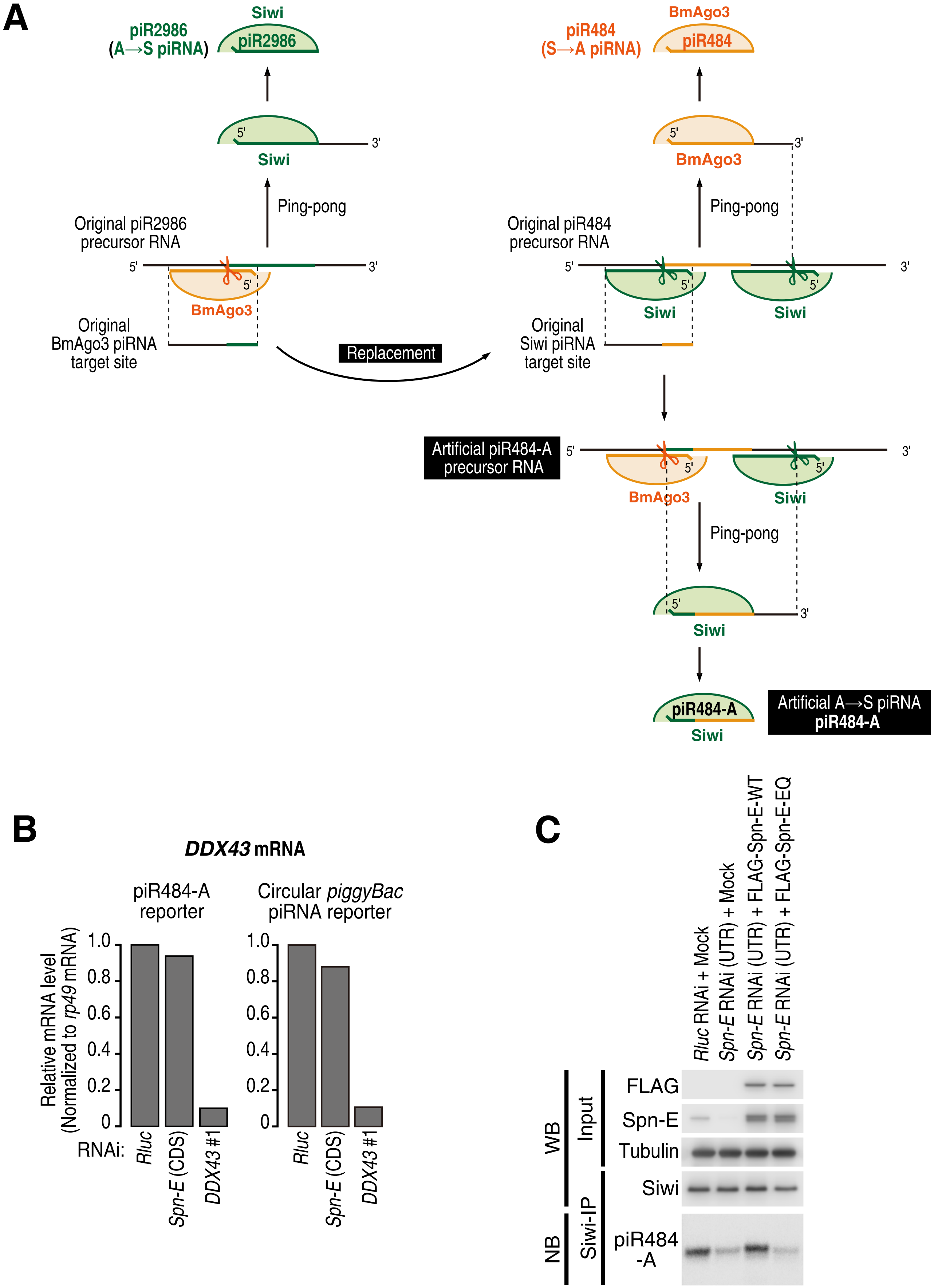
A
Siwi
piR2986
(A→S piRNA)
piR2986
BmAgo3
piR484
(S→A piRNA)
piR484
5'
3'
Siwi
BmAgo3
Ping-pong
Original piR2986 precursor RNA
Original piR484 precursor RNA
BmAgo3
Original BmAgo3 piRNA target site
Replacement
Siwi
Siwi
Original Siwi piRNA target site
Artificial piR484-A precursor RNA
BmAgo3
Siwi
Ping-pong
Siwi
piR484-A
Siwi
Artificial A→S piRNA piR484-A
B
DDX43 mRNA
piR484-A reporter
Circular piggyBac piRNA reporter
Relative mRNA level (Normalized to rp49 mRNA)
1.0
0.8
0.6
0.4
0.2
0
RNAi:
Rluc
Spn-E (CDS)
DDX43 #1
C
Rluc RNAi + Mock
Spn-E RNAi (UTR) + Mock
Spn-E RNAi (UTR) + FLAG-Spn-E-WT
Spn-E RNAi (UTR) + FLAG-Spn-E-EQ
WB
Input
FLAG
Spn-E
Tubulin
Siwi-IP
Siwi
NB
piR484 -A

 © The Author(s)

Figure EV3. Construction of the piR484-A reporter system and Spn-E KD rescue experiment using the piR484-A reporter.

(**A**) Schematic explanation of the construction of the piR484-A reporter. piR484 is an S → A piRNA, and the sequence including the 5′ 10 nt and the upstream region of piR484 was replaced with the corresponding region of piR2986, an A → S piRNA, resulting in an artificial A → S piRNA, piR484-A. The downstream sequence of piR484 that contains a Siwi-bound piRNA target sequence was used without modification. (**B**) Quantitative real-time PCR analysis of the expression of *DDX43* in the reporter experiments in Fig. 3B,D. Relative mRNA expression levels normalized to those of *rp49* are shown. *Rluc*; *Renilla luciferase*, control. (**C**) KD rescue experiment of Spn-E using the piR484-A reporter. Siwi was immunoprecipitated from BmN4 cells co-transfected with the piR484-A reporter plasmid, the FLAG-Spn-E expression plasmid, and dsRNA targeting the *Spn-E* 3′ UTR. Immunoprecipitated Siwi and Siwi-bound piR484-A were detected by western blotting and northern blotting, respectively. *Rluc*; *Renilla luciferase*, control.

© The Author(s)
